# Protective effects of Demethylfuropinnarin on porcine pre-implantation embryos under Tunicamycin-induced oxidative stress and endoplasmic reticulum stress during in vitro culture

**DOI:** 10.1038/s41598-026-38755-6

**Published:** 2026-02-05

**Authors:** Peihong Teng, Shaonan Yu, Feiyang Yang, Ya-nan Zhang, Guifeng Liu, Chuang Li

**Affiliations:** 1 Department of Radiology, China-Japan Union Hospital of Jilin University, Changchun, 130033 China; 2 College of Computer Science and Technology, Jilin University, Changchun, 130012 China; 3 Changchun University of Chinese Medicine, Changchun, 130117 China; 4 College of Modern Agriculture, Changchun Polytechnic University, Changchun, 130022 China; 5 College of Agriculture and Life Science, Chungnam National University, Daejeon, 34314 South Korea

**Keywords:** Antioxidant, Apoptosis, Assisted reproductive technologies (ART), Mitochondrial function, Reactive oxygen species (ROS), Animal physiology, Embryology

## Abstract

Assisted Reproductive Technologies (ART) are essential for addressing infertility and improving reproductive outcomes in both humans and animals. In vitro culture (IVC) supports embryo development outside the body but is often compromised by oxidative and endoplasmic reticulum (ER) stress, leading to impaired development and apoptosis. Demethylfuropinnarin (DMFP), a furocoumarin from *Notopterygium incisum*, has not been previously studied for its biological activity. Given the antioxidant properties of furocoumarins, this study aimed to determine whether DMFP could alleviate tunicamycin (TM)-induced oxidative and ER stress in porcine pre-implantation embryos. Our results show that DMFP significantly reduced reactive oxygen species (ROS) and preserved mitochondrial function. Additionally, DMFP increased Nuclear factor erythroid 2–related factor 2 (Nrf2) expression and decreased Kelch-like ECH-associated protein 1 (Keap1), leading to higher superoxide dismutase (SOD) and catalase (CAT) activity. DMFP treatment also improved cleavage and blastocyst formation rates, reduced apoptosis, and potentially alleviated ER stress, as indicated by lower levels of key ER stress markers. This study concludes that DMFP effectively reduces oxidative stress and may mitigate ER stress, enhancing embryo viability during IVC.

**Supplementary Information:**

The online version contains supplementary material available at 10.1038/s41598-026-38755-6.

## Introduction

Assisted Reproductive Technologies (ART) has become increasingly widespread, enabling individuals unable to conceive naturally to have offspring^1^. Among the various ART techniques, the in vitro culture (IVC) of embryos is a crucial component. IVC is essential for the successful development, selection, and transfer of embryos, directly influencing the success rate and effectiveness of ART^2^. Additionally, IVC technology plays a significant role in animal breeding by enhancing reproductive efficiency, protecting endangered species, and optimizing breeding strategies^3,4^.

However, embryos cultured in vitro exhibit differences compared to those developed in vivo^5,6^. Factors such as light exposure, fluctuations in temperature, humidity, pH, osmotic pressure, and oxygen tension may induce oxidative stress in IVC embryos, presenting significant challenges^7^. During normal cellular metabolism, reactive oxygen species (ROS), including superoxide anions (O2-), hydrogen peroxide (H2O2), and hydroxyl radicals (•OH), are generated^8^. At physiological levels, ROS act as important signaling molecules regulating cellular proliferation, differentiation, and developmental processes^9^. However, when ROS production exceeds the capacity of antioxidant defense systems, this imbalance leads to oxidative stress, which in turn damages cellular DNA and organelles^10^. In embryos, excessive ROS impair cell division, disrupt mitochondrial ATP production, and activate apoptotic pathways such as the Bax/Bcl-2 cascade^11^. These processes can compromise blastocyst formation and developmental potential^12^. Consequently, researchers frequently add antioxidants to the culture medium to counteract or mitigate oxidative stress. Common examples include melatonin, Schisandrin B, vitamin C, which have been reported to improve embryo development by reducing ROS levels and strengthening the antioxidant defense system during embryo culture^13–15^.

The endoplasmic reticulum (ER) is a crucial organelle involved in protein folding, lipid synthesis, and calcium storage^16^. When the ER experiences functional disturbances and accumulates misfolded or unfolded proteins, cells initiate a protective stress response known as ER stress. ER stress, in turn, activates a cellular mechanism called the Unfolded Protein Response (UPR)^17^. The UPR aims to restore normal function by halting protein translation, degrading misfolded proteins, and activating signaling pathways that increase the production of molecular chaperones through three pathways: PERK, IRE1, and ATF6 ^18^. In embryos, if UPR is prolonged or severe, overexpression of C/EBP Homologous Protein (CHOP) inhibits Bcl2 and promotes Bax activity, leading to cellular dysfunction and apoptosis^19^. ER stress may exacerbate oxidative stress in embryos, and conversely, excessive ROS can aggravate ER dysfunction by promoting protein misfolding and calcium imbalance^20^. This bidirectional interaction between ER stress and oxidative stress disrupts cellular homeostasis and constitutes a critical challenge for maintaining embryo viability and developmental potential in ART^16^.

Traditional Chinese Medicine (TCM) has gained increasing global recognition, and herbal medicine research plays a key role in this field^21^. Among these traditional herbs, *Notopterygium incisum* (*N incisum*, Qianghuo) is native to the Qinghai and Sichuan provinces of China, where it thrives at high altitudes^22^. The use of *N incisum* dates back to ancient Chinese medical texts, where it was described for various therapeutic purposes, including reproductive health^23^. Beyond its historical use in classical Chinese medicine, modern phytochemical studies have revealed that extracts from *N. incisum* possess notable antioxidant properties, which provide a scientific basis for investigating its bioactive compounds in biomedical applications^22,24^.

Phytochemical investigations have identified multiple classes of bioactive compounds in N. incisum, with coumarins and their derivatives, including furocoumarins, being of particular interest due to their antioxidant activity^25^. Previous studies have shown that certain coumarins such as imperatorin, isoimperatorin, and osthole can activate the Nuclear factor erythroid 2–related factor 2/Kelch-like ECH-associated protein 1 (Nrf2/Keap1) signaling pathway and upregulating the expression of endogenous antioxidant enzymes, including superoxide dismutase (SOD) and catalase (CAT)^26,27^. Among its bioactive compounds, Demethylfuropinnarin (DMFP) stands out due to its unique chemical structure^28^. DMFP, a furocoumarin, contains the phenolic hydroxyl group and the conjugated double bond, both of which are known to enhance ROS-scavenging ability and antioxidant stability^29^. Additionally, the presence of a lactone ring and a benzofuran moiety in DMFP is considered critical for its redox-regulating activity. These structural elements can stabilize radical intermediates and facilitate electron delocalization, thereby enhancing ROS-scavenging capacity^30^. Similar motifs in related natural products, such as furocoumarins and benzofuran derivatives, have been experimentally shown to activate the Nrf2/Keap1 pathway and promote the expression of antioxidant enzymes, providing mechanistic support for DMFP’s potential antioxidant effects^31,32^. Given the close interplay between oxidative stress and ER stress, compounds with potent antioxidant capacity may also indirectly mitigate ER stress^18,33^. This potential dual action provides the rationale for investigating DMFP’s effects on both oxidative and ER stress in embryos.

The application of tunicamycin (TM) to induce oxidative and ER stress is well documented. TM is an antibiotic that specifically inhibits N-linked glycosylation, resulting in the accumulation of misfolded glycoproteins within the endoplasmic reticulum^34,35^. This disruption of ER function activates the UPR through PERK, IRE1, and ATF6 signaling branches, leading to increased expression of ER stress markers such as GRP78 and CHOP^36^. TM has been widely used in mammalian oocytes and embryos to experimentally model ER stress, where it has been shown to compromise developmental competence and increase apoptosis^37^. In porcine embryos, TM exposure induces protein misfolding, perturbs calcium homeostasis, and triggers oxidative stress in addition to ER dysfunction, collectively impairing early development^13^.

This study aims to investigate whether DMFP can alleviate oxidative and ER stress in porcine embryos during in vitro culture. The findings are expected to provide new insights into improving porcine in vitro reproductive technologies and may also broaden the potential applications of traditional herbal medicines in contemporary biotechnology.

## Materials and methods

Unless otherwise specified, all chemicals and reagents used in this study were purchased from Sigma-Aldrich (St. Louis, MO, USA). All experimental protocols followed our laboratory’s previously published methods^13,38–42^. Detailed protocols for In vitro maturation (IVM), in vitro fertilization (IVF) and in vitro culturing (IVC), staining, Real-time quantitative reverse transcription polymerase chain reaction (RT-qPCR), and Western blotting (WB) are provided in Supplementary File 1. The number of embryos used for each fluorescence assay is indicated directly in the figures. Unless otherwise specified, all chemicals and reagents used in this study were purchased from Sigma-Aldrich (St. Louis, MO, USA).

### Ethics declarations

Ethical standards for research involving the use of ovaries and semen collection were reviewed and approved by the Institutional Animal Care and Use Committee (IACUC) of Chungnam National University (Approval No. 202103 A-CNU-002). All experimental procedures were performed in accordance with relevant guidelines and regulations, including compliance with the ARRIVE (Animal Research: Reporting of In Vivo Experiments) guidelines.

### Isolation and characterization of Demethylfuropinnarin (DMFP) from notopterygium incisum

The underground part of *N incisum* was sourced from the Changchun Traditional Chinese Medicine Market in January 2024. Botanical identification was confirmed by Dr. Ya-nan Zhang from Changchun University of Chinese Medicine. The dried roots and rhizomes of *N incisum* (1 kg) were pulverized and sieved. The powdered material was subjected to reflux extraction using 2.5 L of methanol, performed thrice for 2 h each. The combined methanolic extracts were concentrated under reduced pressure to yield a crude methanol extract. This extract was subsequently dissolved in 2 L of water and extracted five times with 5 L of ethyl acetate. The ethyl acetate layers were pooled, and the solvent was removed under reduced pressure to obtain the ethyl acetate extract. The ethyl acetate extract was decolorized using MCI, followed by gradient elution through a silica gel column using petroleum ether-acetone. DMFP was strictly protected from light exposure and stored at 4 °C. After purification, the pale-yellow crystalline compound was identified as DMFP based on comparison of its melting point, ESI-MS, and ^1H-NMR data with those reported for compound 6 by Kozawa et al. (1983) and compound 18 by Liu et al. (2012)^43,44^.

HPLC chromatographic analysis was performed using a C18 column (150 mm × 4.6 mm, 5 μm) with a mobile phase consisting of methanol (A) and acetic acid aqueous solution (61:4, B). The elution gradient was programmed from 20% A to 100% A over 15 min at a flow rate of 1.2 mL/min. The column temperature was maintained at 30 °C, and detection was conducted at 315 nm. The injection volume was 10 µL. The chromatogram revealed a major peak at approximately 8.70 min corresponding to the purified DMFP, and a minor peak at 4.32 min corresponding to a residual impurity (Supplementary Fig. 1). By comparing the areas of the main peak and the minor peak, the purity of DMFP was determined to be greater than 95%, and the spectroscopic data are as follows:

Melting point: 231°C; ESI-MS m/z: 271 [M+]; ˆ1H-NMR spectrum (DMSO-d6, 600 MHz): *δ* 11.00 (1H, s, OH), 8.25 (1H, d, J = 9.8 Hz, H-4), 7.87 (1H, d, J = 2.3 Hz, H-2’), 7.16 (1H, d, J = 2.2 Hz, H-3’), 6.29 (1H, dd, J = 17.4, 10.4 Hz, H-2”), 6.24 (1 H, d, J = 9.7 Hz, H-3), 4.92 (1 H, d, J = 10.1 Hz, H-3”*α*), 4.87 (1 H, d, J = 17.8 Hz, H-3”*β*), and 1.74 (6 H, s).

### In vitro maturation (IVM), in vitro fertilization (IVF) and in vitro culturing (IVC)

Briefly, ovaries from prepubertal gilts were collected from a local slaughterhouse and transported to the laboratory at 37 °C. Cumulus-oocyte complexes (COCs) were aspirated from follicles 3 to 8 mm in diameter, ensuring each COC had at least three layers of compact cumulus cells surrounding a homogenous, granular ooplasm. Selected COCs were washed and incubated in IVM medium at 38.5 °C in a humidified atmosphere with 100% humidity and 5% CO_2_ for 22 h, followed by a further 22 h in hormone-free maturation medium (IVM medium without eCG and hCG). After 44 h, maturation rates of oocytes were observed. Oocytes with expelled polar bodies were selected and washed in Modified Tris Buffered Medium (mTBM). Boar sperm in a commercial extender was obtained from Darby Genetics Inc. (Seodongdearo Iljuk-myun Anseong Gyeonggi-do Republic of Korea) and transported to the laboratory at 17–18 °C. Only semen samples with ≥ 80% total motility and ≤ 15% morphologically abnormal spermatozoa were used. Following centrifugation at 500 × g for 4 min, the supernatant was removed. The sperm was then diluted in mTBM to achieve a final concentration of 0.25 × 10^6^ sperm/mL and subsequently co-incubated with oocytes for 6 hours. Embryos were washed and cultured in porcine zygote medium 3 (PZM-3) with 100% humidity, 5% O_2_, and 5% CO_2_, with cleavage and blastocyst rates assessed at 48 and 168 h, respectively. The calculation of blastocyst rates included only expanded, hatching, and hatched blastocysts, as illustrated in Supplementary Fig. 2.

### DMFP and Tunicamycin (TM) treatment

DMFP and TM treatment was performed throughout the entire 7-day IVC phase. DMFP and TM (Sigma-Aldrich, T7765) were dissolved in dimethyl sulfoxide (DMSO) to prepare a stock solution of 1 mg/mL. The final DMSO concentration in all groups was adjusted to 0.1% (v/v), which was used as the vehicle for dissolving DMFP and TM. This concentration was selected based on the study by Lucas et al. (2021), which demonstrated that ≤ 0.1% DMSO does not affect porcine embryo development^45^. DMFP and TM were added to the PZM-3 medium at designated concentrations as described in the experimental design.

### Analysis of reactive oxygen species (ROS) and glutathione (GSH) levels in blastocysts

ROS and Glutathione (GSH) levels were measured using the Image-IT™ LIVE Green ROS Detection Kit (Invitrogen, Carlsbad, CA, USA) and Invitrogen Cell Tracker™ Blue CMHC (Invitrogen, Carlsbad, CA, USA). Blastocysts were washed three times in 0.1% phosphate-buffered saline containing polyvinyl alcohol (PBS/PVA), incubated with 25 *µ*M H2DCF-DA or 10 *µ*M CMF2HC dye in 0.1% PBS/PVA at 38.5 °C for 30 min, and washed before detecting fluorescence (ROS: 460 nm; GSH: 370 nm).

### Analysis of mitochondrial function and endoplasmic reticulum (ER) distribution in blastocysts

Mitochondrial functions and ER distribution in blastocysts were assessed using MitoTracker™ Red CMXRos and ERTracker™ (Thermo Fisher Scientific, Waltham, MA, USA). Blastocysts were washed with 0.1% PBS/PVA and stained with PBS/PVA containing 250 nM MitoTracker™ Red CMXRos or 200 nM ERTracker dye at 38.5 °C for 30 min. They were fixed in 4% PBS/PFA for 30 min at room temperature (24–25 °C), then washed with PBS, and fluorescence (580 nm) was detected.

### Analysis of mitochondrial membrane potential (MMP) levels in blastocysts

Mitochondrial membrane potential (MMP) levels were estimated using the MitoProbe™ JC-1 Assay Kit (Invitrogen, Carlsbad, CA, USA). Blastocysts were washed with 0.1% PBS/PVA, and incubated with 2 *µ*M JC-1 in PBS at 38.5 °C and 5% CO_2_ for 30 min. Fluorescence from red (J-aggregates, 580 nm) and green (J-monomers, 460 nm) were detected, and MMP levels were calculated as the ratio of red to green fluorescence intensity.

### Analysis of apoptosis levels in blastocysts

Apoptosis in blastocysts was assessed using the TUNEL assay kit (In Situ Cell Death Detection Kit, TMR Red, Roche Diagnostics, Mannheim, Germany). Blastocysts were washed in 0.1% PBS/PVA, fixed in 4% phosphate-buffered saline containing paraformaldehyde (PBS/PFA) for 1 h, permeabilized with 1% Triton X-100 for 5 min at 4 °C, and incubated in TUNEL reaction medium at 38.5 °C for 1 h. After washing and Hoechst 33,342 staining, apoptosis (580 nm) and total cell numbers (370 nm) were detected.

### Fluorescence detection and quantification in blastocysts

All fluorescence was detected using a Leica DMi8 Fluorescence Microscope (Leica, Wetzlar, Germany). All fluorescence intensity analyses were conducted using Image J 1.51 software (National Institutes of Health, Bethesda, MD, USA) and reported as relative fluorescence intensities, with the mean intensity of the control group normalized to 1.

### Analysis of superoxide dismutase (SOD) and catalase (CAT) activity levels in blastocysts

SOD and CAT activity levels were measured using OxiSelect™ Catalase Activity Assay Kit and OxiSelect™ Superoxide Dismutase Activity Assay Kit (Cell Biolabs Inc., San Diego, CA, USA). Fifty blastocysts from each group were used, and all procedures followed previously published methods from our laboratory^38–40^. SOD and CAT activities were assessed by measuring absorbance at 490 and 520 nm, respectively, using an ELISA reader (EPOCH, BioTek Instruments Inc, Winooski, VT, USA). Results are presented as relative data, with the mean of the control group set to 1 and without units.

### Real-time quantitative reverse transcription polymerase chain reaction (RT-qPCR)

Total RNA was extracted from each group of blastocysts using the RNAqueous™ Micro Kit (Thermo Fisher Scientific, Waltham, MA, USA). RNA concentration and purity were measured using a BioSpec-nano spectrophotometer (Shimadzu Biotech, Kyoto, Japan). Only samples with an A260/A280 ratio between 1.8 and 2.1 were used for cDNA synthesis. Complementary DNA (cDNA) was synthesized using the Maxime™ PreMix (iNtRON, Seongnam, Jungwon, South Korea). Quantitative PCR amplification was carried out using the CFX96 Real-Time PCR Detection System (Bio-Rad Laboratories, Hercules, CA, USA) with PowerUp SYBR Green Master Mix (Applied Biosystems, Foster City, CA, USA). The mRNA levels of Spliced X-box Binding Protein 1 (sXBP1), Glucose-Regulated Protein 78 (GRP78), Activating Transcription Factor 4 (ATF4), C/EBP Homologous Protein (CHOP), Bcl-2-associated X protein (Bax), and B-cell lymphoma 2 (Bcl2) were measured. Primers used in this study are listed in Supplementary Table 1. The relative mRNA expression levels of other genes were normalized to the relative mRNA expression level of Glyceraldehyde 3-phosphate dehydrogenase (GAPDH), which has been previously validated as a stable reference gene in early porcine embryos^13,46^. Relative expressions of each gene were quantified using the 2^−∆∆*Ct*^ method.

### Western blot (WB)

The anti- Nrf2 antibody (BS-1074R) and anti-Keap1 antibody (BS-3648R) were purchased from Thermo Fisher Scientific (Waltham, MA, USA); while the anti-*β*-actin antibody was obtained from Sigma-Aldrich (St. Louis, MO, USA). The secondary antibody (goat anti-rabbit) was purchased from Santa Cruz Biotechnology (Santa Cruz, CA, USA). Briefly, blastocysts were lysed in RIPA buffer (ELPIS Bio, Eunpyeong-gu, Seoul, South Korea), with a minimum number of 40 per group. Proteins were then precipitated using acetone and re-suspended in TEN buffer. Protein concentrations were measured using a Bio-Rad protein assay kit. Subsequently, proteins were separated by SDS-PAGE (10% gel) and transferred to PVDF membranes (Bio-Rad Laboratories, Hercules, CA, USA). After incubation with the appropriate primary and secondary antibodies, protein bands were visualized using enhanced chemiluminescence (Amersham Biosciences, Arlington Heights, IL, USA) and examined using the Alliance Q9 Advanced imaging system (UVItec Ltd., Cambridge, UK). Uncropped full-length blot images are provided in Supplementary Fig. 6.

### Experimental design

To assess the protective effects of DMFP on porcine embryos, the developmental capacity, ROS and GSH levels, SOD and CAT activity levels, protein expression levels of Nrf2 and Keap1, mitochondrial function, MMP levels, ER distribution, mRNA expression of sXBP1, ATF4, GRP78, CHOP, Bcl2, and Bax, and apoptosis rates of blastocysts in control (1 mL/L, 0.1% v/v DMSO treatment), DMFP (1 mg/L DMFP treatment), TM (0.1 mg/L TM treatment), and DMFP + TM (1 mg/L DMFP and 0.1 mg/L TM co-treatment) groups were measured. Preliminary experiments comparing untreated embryos with those exposed to 0.1% DMSO revealed no significant differences in cleavage and blastocyst rates (data not shown). Therefore, embryos treated with 0.1% DMSO were used as the vehicle control in this study. The concentration of DMFP was selected based on results from our preliminary dose-response experiment (see Supplementary Figs. 3 and 4 and Supplementary Table 2 for details). The TM concentration was adopted from our previously published study^13,37^.

### Statistical analysis

All data are presented as mean ± SD. The number of replicates and sample sizes for each experiment are indicated in the corresponding figures and tables. Percentage data were analyzed using a beta regression model. Continuous data were tested for normality and homogeneity of variance, and if assumptions were met, they were analyzed using one-way ANOVA followed by Tukey’s post-hoc test or Student’s t-test. Statistical analyses were performed using R 4.3.2. Significance was set at *P <* 0.05.

Result.

### Effects of DMFP and TM on the developmental potential of porcine embryos

**Fig. 1:**
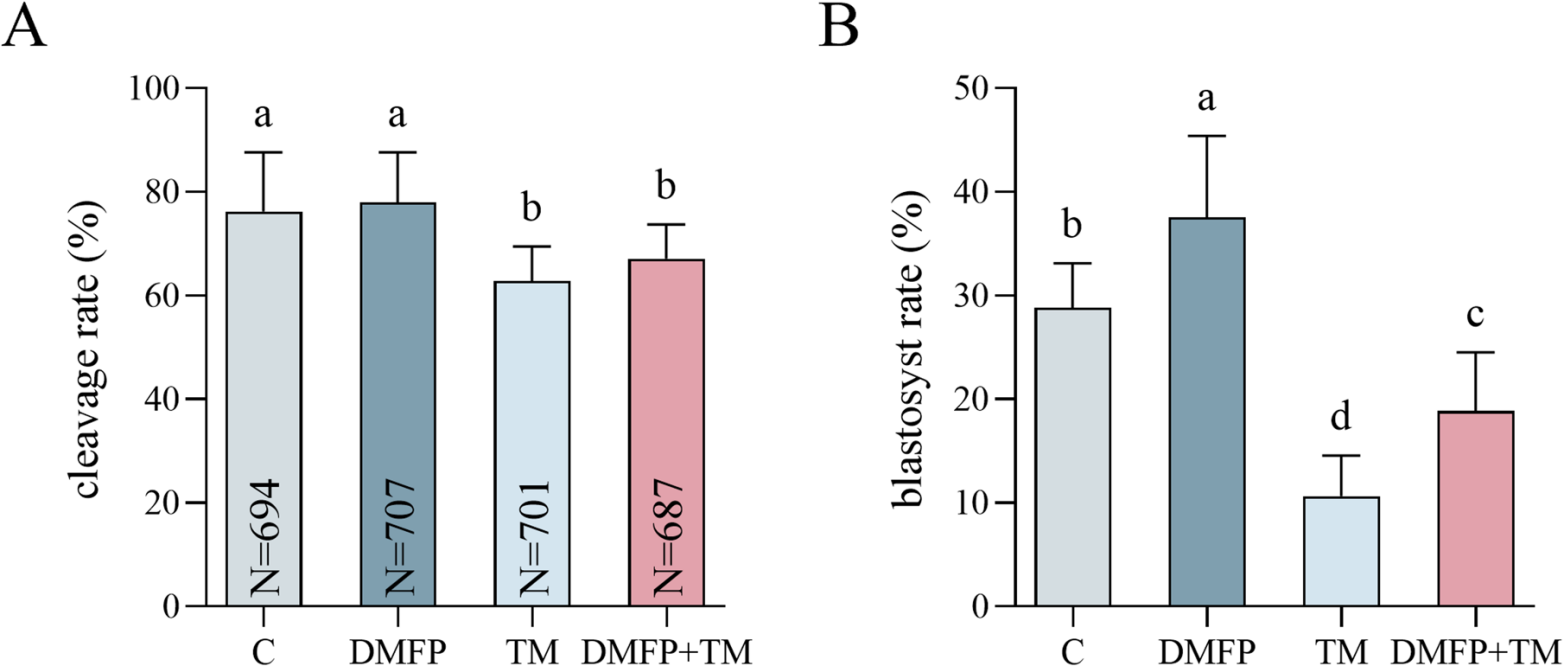
Effects of DMFP and TM on the developmental potential of porcine embryos. The cleavage rate (**A**) and blastocyst rate (**B**) of embryos from the control group, DMFP group (treated with 1 mg/L DMFP), TM group (treated with 0.1 mg/L TM), and DMFP + TM group (co-treated with 1 mg/L DMFP and 0.1 mg/L TM). Sample sizes (N) are indicated directly within each bar of the figure. A total of 15 independent experimental replicates were performed. Different superscripts indicate statistical differences within experimental groups (*P <* 0.05).

Cleavage and blastocyst formation rates were analyzed after exposure to 1 mg/L DMFP and 0.1 mg/L TM, as presented in Fig. 1 and Supplementary Table 3. The developmental status of embryos in each group is shown in Supplementary Fig. 5. The cleavage rate in the DMFP group was significantly higher than those in TM and DMFP + TM groups (Fig. 1A, P *<* 0.05), while the cleavage rate in the TM group was significantly lower than in the control group (Fig. 1A, P *<* 0.05). The blastocyst rate of DMFP group was significantly higher than other groups, with the TM group being significantly lower than other groups (Fig. 1B, P *<* 0.05). The blastocyst rate of the DMFP + TM co-treatment group was significantly lower than the control group (Fig. 1B, P *<* 0.05).

### Effects of DMFP and TM on oxidative stress within Porcine blastocysts

**Fig. 2:**
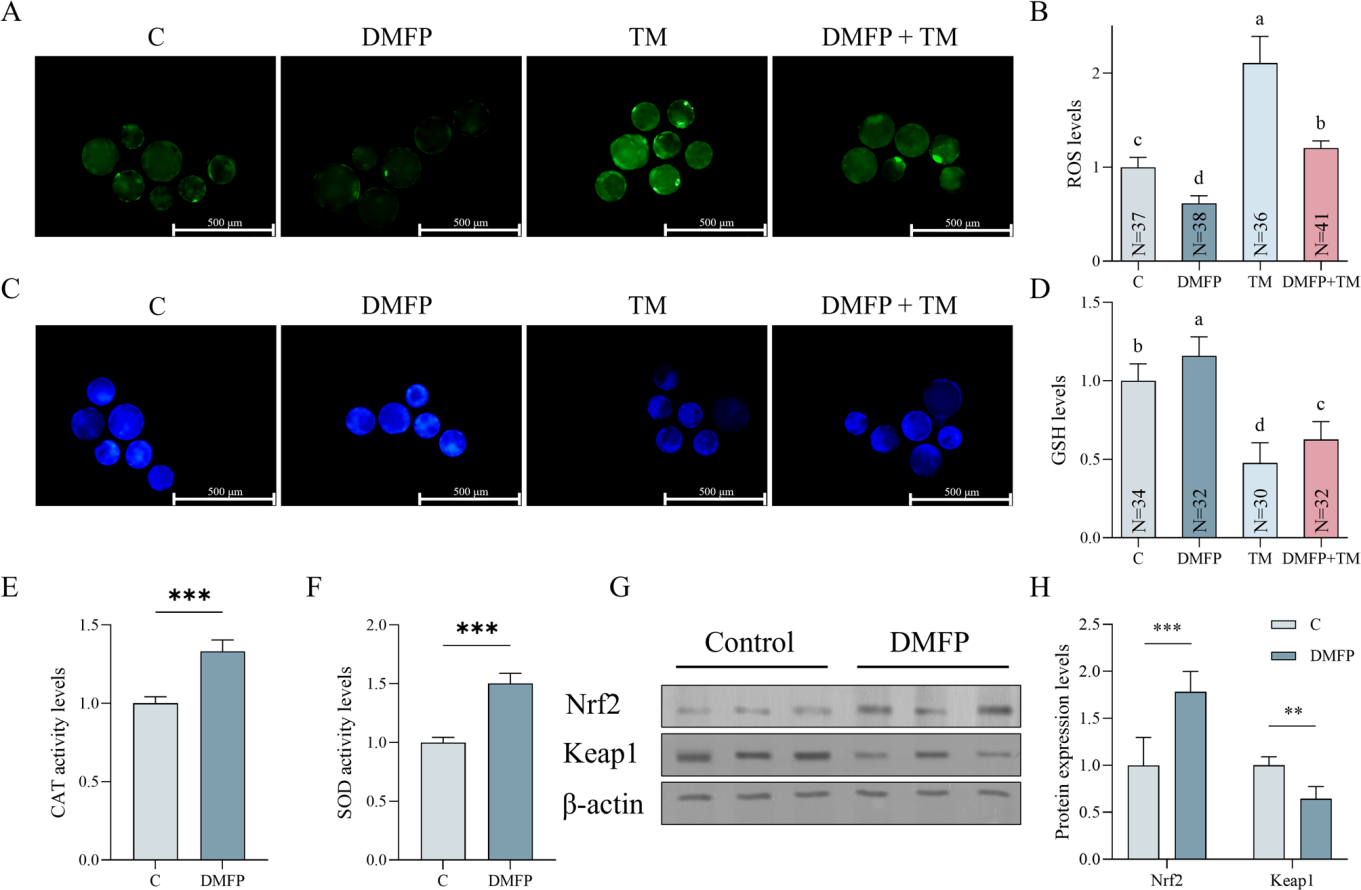
Effects of DMFP and TM on oxidative stress within porcine blastocysts. The fluorescence micrograph (**A**) and fluorescence intensity (**B**) of ROS, and fluorescence micrograph (**C**) and fluorescence intensity (**D**) of GSH of blastocysts from the control group, DMFP group (treated with 1 mg/L DMFP), TM group (treated with 0.1 mg/L TM), and DMFP + TM group (co-treated with 1 mg/L DMFP and 0.1 mg/L TM); the activity levels of CAT (**E**), SOD (**F**) and western blot images (**G**) and quantification (**H**) of Nrf2 and Keap1 of blastocysts from the control group and DMFP group (treated with 1 mg/L DMFP). Sample sizes (N) are indicated directly within each bar of the figure. For ROS and GSH levels, 4 independent experimental replicates were performed. For SOD and CAT activities, 9 independent replicates were performed, while for WB of Nrf2 and Keap1, 9 independent replicates were conducted. Different superscripts indicate statistical differences within experimental groups (*P <* 0.05), “**” indicates statistical significance (*P <* 0.01), “***” indicates statistical significance (*P <* 0.001).

The ROS levels in blastocysts across different groups are depicted in Fig. 2. The ROS levels in the DMFP group were significantly lower compared to other groups, while the ROS levels in the TM group were significantly higher than in other groups (Fig. 2B, P *<* 0.05). The ROS levels in the DMFP + TM co-treatment group were significantly higher compared to the control group (Fig. 2B, P *<* 0.05). As for the GSH levels, the DMFP group was significantly higher than other groups, the TM group was significantly lower than other groups, and the DMFP + TM co-treatment group was significantly lower than the control group (Fig. 2D, P *<* 0.05). The activities of CAT and SOD and the protein expression of Nrf2 in blastocysts treated with 1 mg/L DMFP were significantly higher than in the control group (Figs. 2E, F, and H, *P <* 0.05). Keap1 protein expression in blastocysts treated with 1 mg/L DMFP was significantly lower than in the control group (Figs. 2H, P *<* 0.05).

### Effects of DMFP and TM on ER stress within porcine blastocysts

**Fig. 3:**
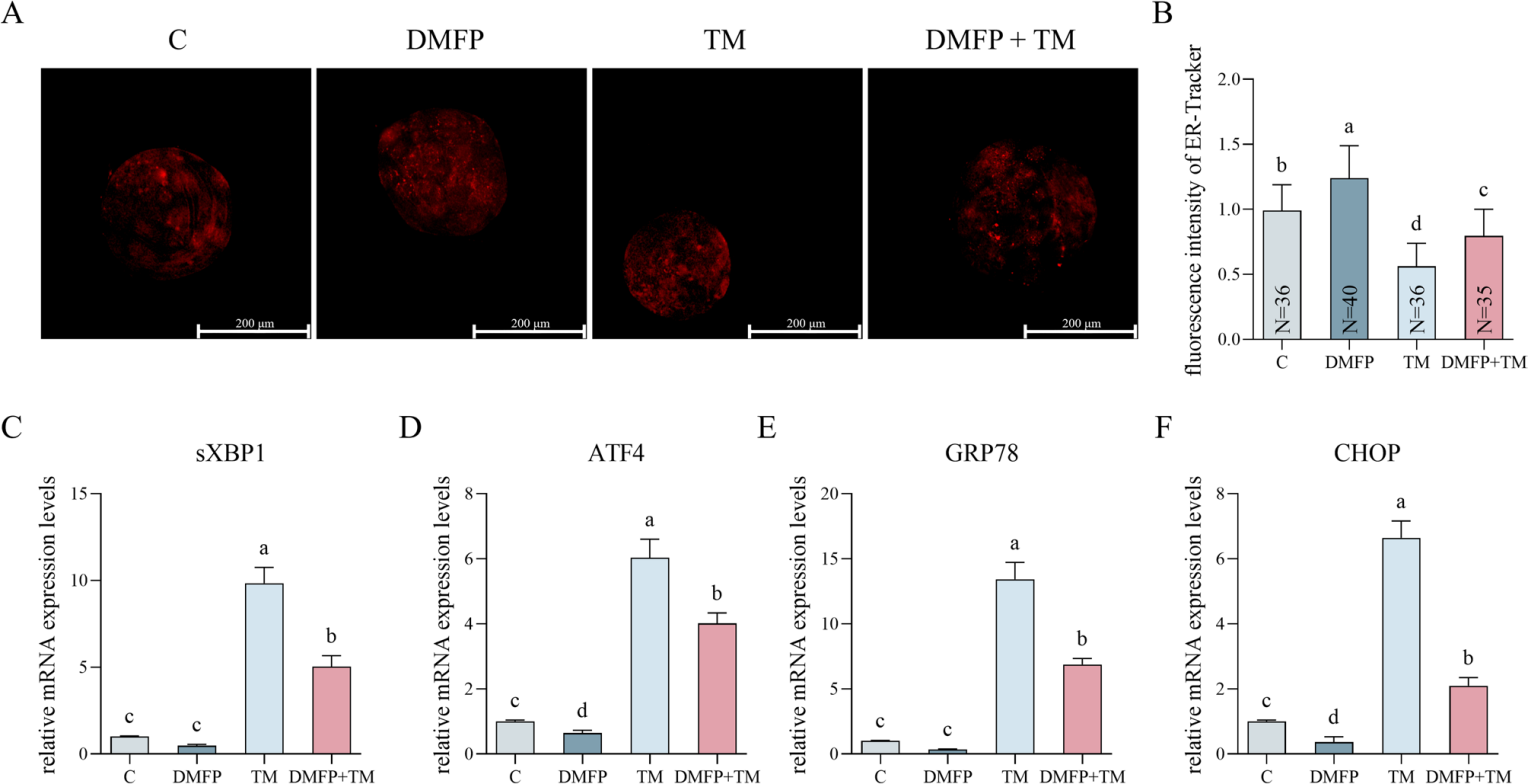
Effects of DMFP and TM on the ER stress within porcine blastocysts. The ER-Tracker staining fluorescence micrograph (**A**), fluorescence intensity (**B**), and the mRNA expression levels of the sXBP1 (**C**), ATF4 (**D**), GRP78 (**E**) and CHOP (**F**) genes of blastocysts from the control group, DMFP group (treated with 1 mg/L DMFP), TM group (treated with 0.1 mg/L TM), and DMFP + TM group (co-treated with 1 mg/L DMFP and 0.1 mg/L TM). Sample sizes (N) are indicated directly within each bar of the figure. For ER-Tracker, 6 independent experimental replicates were performed. For mRNA expression levels of sXBP1, ATF4, GRP78, and CHOP, 9 independent replicates were conducted. Different superscripts indicate statistical differences within experimental groups (*P <* 0.05).

The ER distribution in blastocysts treated with 1 mg/L DMFP and 0.1 mg/L TM is presented in Fig. 3. The fluorescence intensity of ER in the DMFP group was significantly higher than in other groups (Fig. 3B, P *<* 0.05), while in the TM group, it was significantly lower than in other groups (Fig. 3B, P *<* 0.05). The DMFP and TM co-treatment group was lower than the control group (Fig. 3B, P *<* 0.05). The trend of the relative mRNA expression levels of sXBP1, ATF4, GRP78, and CHOP in the groups showed the opposite trend as the fluorescence intensity (Fig. 3C, D, E, F).

### Effects of DMFP and TM on mitochondria within porcine blastocysts

**Fig. 4:**
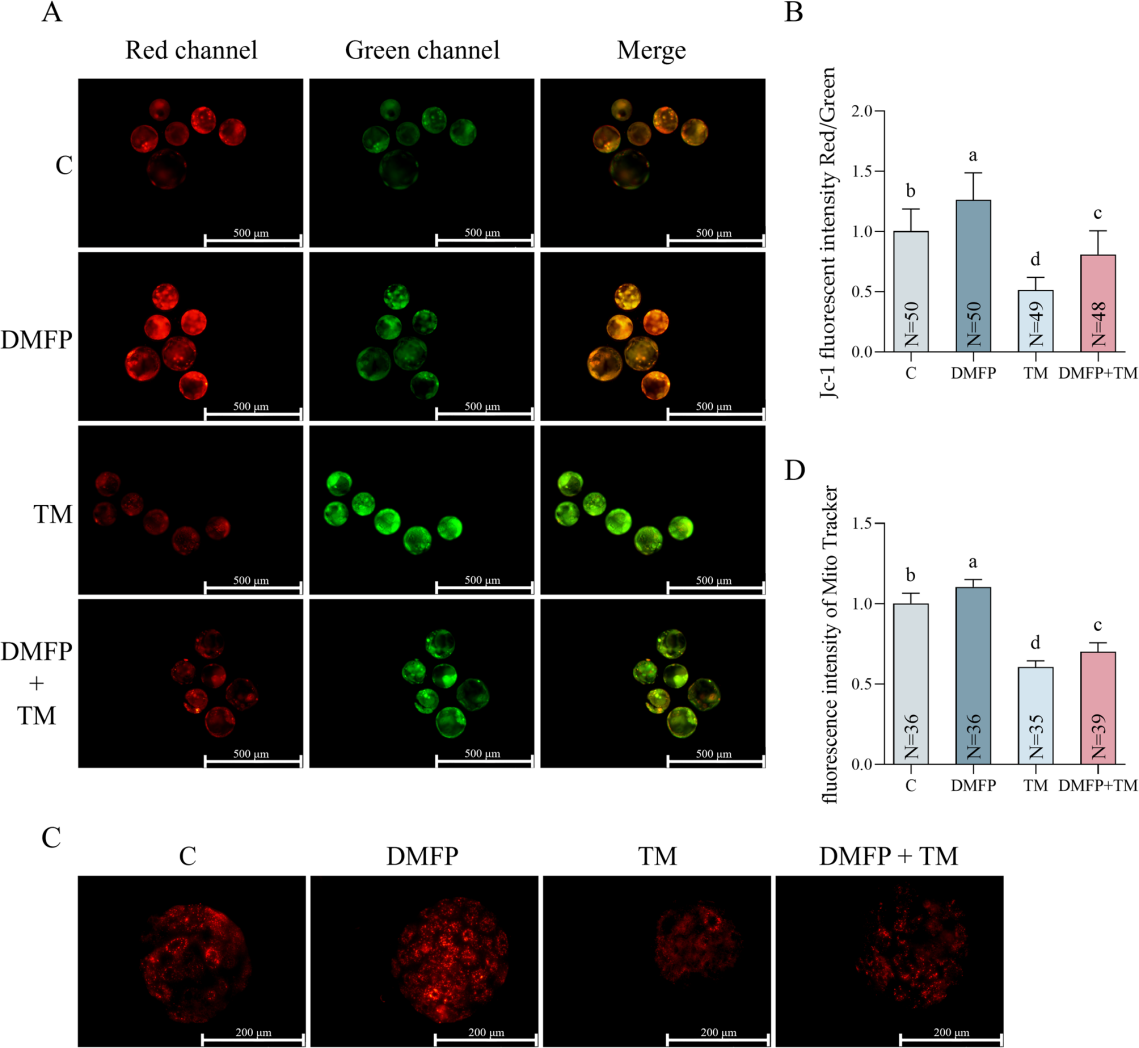
Effects of DMFP and TM on mitochondria damage within porcine blastocysts. The fluorescence micrograph (**A**) and fluorescence intensity rate (**B**) of JC-1, and fluorescence micrograph (**C**) and fluorescence intensity (**D**) of Mito Tracker of blastocysts from the control group, DMFP group (treated with 1 mg/L DMFP), TM group (treated with 0.1 mg/L TM), and DMFP + TM group (co-treated with 1 mg/L DMFP and 0.1 mg/L TM). Sample sizes (N) are indicated directly within each bar of the figure. For JC-1, 8 independent experimental replicates were performed. For Mito Tracker, 6 independent replicates were conducted. Different superscripts indicate statistical differences within experimental groups (*P <* 0.05).

After staining with JC-1 and MitoTracker, MMP and mitochondrial function in porcine blastocysts treated with all groups were assessed, as shown in Fig. 4. The MMP of the DMFP group was significantly higher compared to all other groups (Fig. 4B, P *<* 0.05), and the TM group was significantly lower (Fig. 4B, P *<* 0.05). In the DMFP and TM co-treatment group, MMP was significantly lower compared to the control group (Fig. 4B, P *<* 0.05). The significant differences in mitochondrial function among groups mirrored the results of MMP (Fig. 4D).

### Effects of DMFP and TM on apoptosis levels within porcine blastocysts

**Fig. 5:**
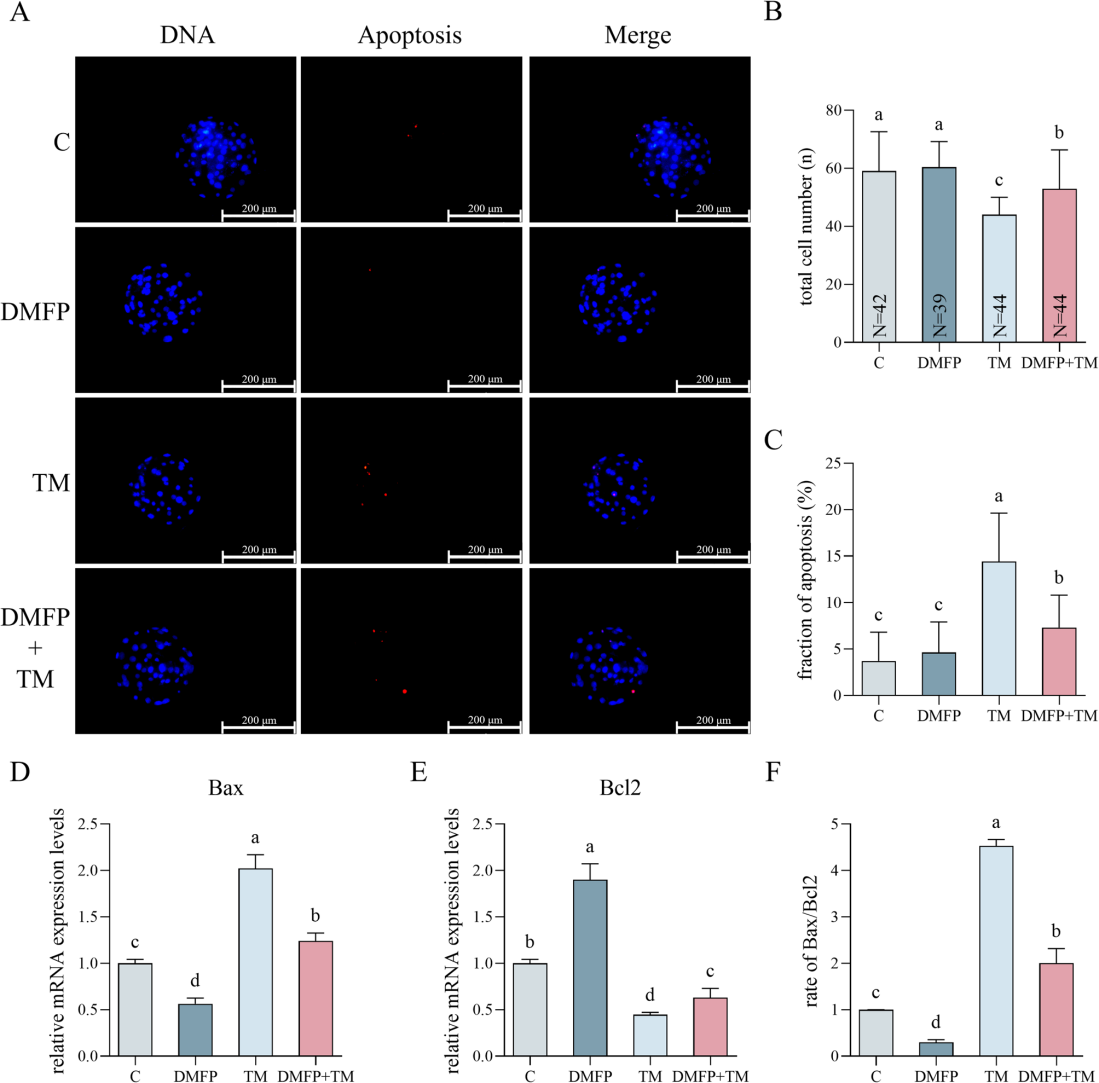
Effects of DMFP and TM on apoptosis levels within porcine blastocysts. The TUNEL and Hoechst 33,342 staining fluorescence micrograph (**A**), total cell number (**B**), fraction of apoptosis (**C**), and the mRNA expression levels of the Bax (**D**) and Bcl2 (**E**) genes and the ratio of Bax/Bcl2 (**F**) of blastocysts from the control group, DMFP group (treated with 1 mg/L DMFP), TM group (treated with 0.1 mg/L TM), and DMFP + TM group (co-treated with 1 mg/L DMFP and 0.1 mg/L TM). Sample sizes (N) are indicated directly within each bar of the figure. For TUNEL, 7 independent experimental replicates were performed. For mRNA expression of Bax and Bcl2, 9 independent replicates were conducted. Different superscripts indicate statistical differences within experimental groups (*P <* 0.05).

To evaluate the apoptosis levels of embryos subjected to DMFP and TM treatments post-IVF, both total cell numbers and apoptotic cells in blastocysts were analyzed, as shown in Fig. 5A. Following TM treatment, TCN in both the TM-only and DMFP + TM co-treatment groups was significantly lower compared to the control group and DMFP group (Fig. 5B, P *<* 0.05). Regarding apoptotic rates, the TM group displayed a significantly higher number than the other groups (Fig. 5C, P *<* 0.05), and the rate in the DMFP + TM group was significantly higher than in the control group and DMFP group (Fig. 5C, P *<* 0.05). Additionally, the mRNA expression of Bax in blastocysts form the DMFP group was significantly lower than other groups (Fig. 5D, P *<* 0.05), with TM being significantly higher than other groups (Fig. 5D, P *<* 0.05), and DMFP + TM was significantly higher than the control group (Fig. 5D, P *<* 0.05). The mRNA expression of Bcl2 in blastocysts form the DMFP group was significantly higher than other groups (Fig. 5E, P *<* 0.05), with TM being significantly lower than other groups (Fig. 5E, P *<* 0.05), and DMFP + TM was significantly lower than control group (Fig. 5E, P *<* 0.05). The rate of Bax and Bcl2 mRNA expression levels in the TM group was significantly higher than in other groups (Fig. 5F, P *<* 0.05), with DMFP group being significantly lower than other groups (Fig. 5F, P *<* 0.05). In the DMFP + TM group, it was lower than in the control groups (Fig. 5F, P *<* 0.05).

## Discussion

This study validates the biological activity of DMFP and examines its antioxidant effects in early porcine embryos derived from IVF under TM-induced stress. In this study, we selected a DMSO-treated (0.1% v/v) group as the control because both DMFP and TM were dissolved in DMSO. Previous studies have demonstrated that low concentrations of DMSO do not affect porcine embryo development^45^. Consistently, our preliminary comparison between untreated and 0.1% DMSO-treated embryos revealed no differences in cleavage and blastocyst formation rates (data not shown). Nevertheless, the absence of a true no-treatment group in the main experimental design remains a limitation. We first conducted a preliminary evaluation of different DMFP concentrations on embryonic development. The results showed that 1 mg/L DMFP significantly enhanced embryonic development, likely due to its antioxidant properties, whereas 100 mg/L DMFP negatively affected embryonic viability. This adverse effect may be attributed to excessively low ROS levels induced by the high concentration of DMFP, which disrupted redox homeostasis and triggered reductive stress, ultimately impairing embryonic development^47,48^. To our knowledge, this is the first study to report such antioxidant and stress-protective effects of DMFP in mammalian embryos.

To further elucidate the protective effects of DMFP, we established a damage model by using TM to induce oxidative and ER stress in porcine embryos. Our results confirmed that TM exposure significantly impaired embryonic development, elevated ROS levels, and activated both oxidative and ER stress pathways, as evidenced by increased expression of GRP78, CHOP, and sXBP1. These findings are consistent with previous studies suggesting that TM disrupts ER homeostasis, which leads to the accumulation of unfolded proteins and activation of the UPR, thereby inducing ER stress^49,50^. In porcine embryos, this stress cascade has been associated with mitochondrial dysfunction and apoptosis^51^. In our model, TM treatment reduced blastocyst formation rates, increased apoptosis, and compromised mitochondrial membrane potential, supporting its detrimental effect on early embryonic competence.

GSH is a tripeptide composed of glutamate, cysteine, and glycine^52^. DMFP treatment reduced ROS levels and enhanced GSH concentrations, suggesting its role in alleviating oxidative stress. SOD2 and CAT are two antioxidant enzymes regulated by Nrf2. Within cells, SOD2 converts superoxide radicals (O2•-) into hydrogen peroxide and oxygen, while CAT breaks down hydrogen peroxide into water and oxygen^53^. Increased expression and activity of SOD2 and CAT further support DMFP’s potential to enhance antioxidant defenses. However, it remains unclear whether DMFP directly activates the Nrf2/Keap1 pathway or influences it indirectly via other mechanisms. Given the strong interplay between oxidative and ER stress, we examined markers of ER stress. DMFP treatment mitigated these effects, indicating its protective role against ER stress. While these results suggest that DMFP reduces ER stress, it remains to be determined whether this effect is a direct action or a consequence of reduced oxidative stress. Mitochondrial dysfunction, characterized by decreased MMP and ATP synthesis, is a major consequence of excessive ROS^54,55^. Our results demonstrated that DMFP treatment restored MMP and reduced apoptosis. This highlights DMFP’s role in preserving mitochondrial integrity and promoting embryonic survival.

This antioxidant effect of DMFP may be attributable to its structural similarity to other well-characterized furocoumarins and coumarin derivatives, such as imperatorin, isoimperatorin, and osthole. These compounds have been reported to alleviate oxidative stress by modulating the Nrf2/Keap1 pathway and enhancing endogenous antioxidant enzyme expression, including SOD and CAT. Under basal conditions, Nrf2 is sequestered in the cytoplasm by Keap1 and targeted for degradation. Upon exposure to oxidative stress or certain electrophilic compounds, Keap1 undergoes conformational changes, leading to the stabilization and nuclear translocation of Nrf2. Once in the nucleus, Nrf2 binds to antioxidant response elements (ARE) in the promoter regions of target genes, promoting the transcription of antioxidant enzymes such as SOD2 and CAT. Several coumarins, including imperatorin and osthole, have been demonstrated to activate this pathway by disrupting Keap1-Nrf2 interactions or modifying Keap1 cysteine residues^24,26^. Structurally, DMFP contains a conjugated aromatic system with a phenolic hydroxyl group and a benzofuran-lactone core, which are also present in these related compounds and are thought to contribute to their ROS-scavenging capacity and redox-regulating activity^30,31^. The resemblance in both chemical features and biological function supports the hypothesis that DMFP may act through similar mechanisms to reinforce the embryo’s antioxidant defenses.

To our knowledge, evidence of such effects in embryos has been limited, and this is the first study to demonstrate that DMFP exerts antioxidant and stress-protective roles in mammalian embryos. Although our preliminary research demonstrates the protective effects of DMFP on porcine pre-implantation embryos, DMFP did not completely eliminate the negative impacts of TM, including impaired developmental competence. This limitation may be attributed to several factors. First, the current study primarily focused on oxidative stress and ER stress pathways without thoroughly investigating other potential mechanisms, such as inflammatory responses or metabolic alterations, which could also contribute to TM-induced damage. Second, the in vitro nature of this study limits the direct generalization of our findings to in vivo conditions. While systemic interactions in vivo may influence the efficacy of DMFP, no in vivo evidence is currently available, and further studies are required to evaluate its effects under physiological conditions. Finally, while DMFP mitigated TM-induced oxidative and ER stress to a certain extent, the residual stress levels suggest that higher or more targeted concentrations of DMFP, or its combination with other antioxidants, may be necessary to achieve more comprehensive protection. Future studies should address these limitations by incorporating a broader scope of analyses, exploring alternative pathways, and validating DMFP’s effects in vivo to optimize its application in reproductive technologies.

Although several antioxidants such as melatonin and vitamin C have well-documented efficacy in embryo culture^14,15^, our aim was to investigate DMFP as a novel candidate compound. DMFP is a unique furocoumarin derivative isolated from *N incisum*, and its biological activities have not been previously studied in the context of embryonic development. While its protective effects were partial compared with some established antioxidants, evaluating such unexplored compounds expands the repertoire of potential agents for reproductive biotechnology and contributes to understanding the pharmacological potential of traditional herbal medicine–derived molecules. Importantly, this work should be regarded as a proof-of-concept study demonstrating the feasibility of applying DMFP—a structurally unique furocoumarin containing a phenolic hydroxyl group and a benzofuran-lactone core—to embryo culture systems. These structural motifs are known to confer strong redox-regulating capacity and potential synergistic interactions with endogenous antioxidant pathways. Thus, beyond its immediate findings, this study broadens the spectrum of bioactive small molecules that can be explored for improving embryo viability and redox homeostasis in vitro.

## Conclusions

These results suggest that DMFP, at a concentration of 1 mg/L, may exert a protective effect in porcine embryos by modulating redox homeostasis and ER stress pathways, thereby partially alleviating the oxidative and ER stress induced by 0.1 mg/L TM. This study demonstrates the potential of DMFP, a traditional herbal compound, as a candidate for alleviating oxidative and ER stress in porcine embryos, and provides mechanistic insights that may inform future applications of natural products in reproductive biotechnology and human ART.

## Supplementary Information

Below is the link to the electronic supplementary material.

Supplementary Material 1

## Data Availability

The datasets used and/or analyzed during the current study are available from the corresponding author upon reasonable request.

## Abbreviations

ARE: Antioxidant Response Element
ART: Assisted Reproductive Technologies
ARRIVE: Animal Research: Reporting of In Vivo Experiments
ATF4: Activating Transcription Factor 4
ATF6: Activating Transcription Factor 6
Bax: Bcl-2-associated X protein
Bcl2: B-cell lymphoma 2
CAT: Catalase
cDNA: Complementary DNA
CHOP: C/EBP Homologous Protein
COCs: Cumulus–Oocyte Complexes
DMSO: Dimethyl Sulfoxide
DMFP: Demethylfuropinnarin
ELISA: Enzyme-Linked Immunosorbent Assay
ESI-MS: Electrospray Ionization Mass Spectrometry
ER: Endoplasmic Reticulum
GAPDH: Glyceraldehyde 3-phosphate dehydrogenase
GRP78: Glucose-Regulated Protein 78
GSH: Glutathione
IACUC: Institutional Animal Care and Use Committee
IVF: In Vitro Fertilization
IVC: In Vitro Culture
IVM: In Vitro Maturation
IRE1: Inositol-Requiring Enzyme 1
Keap1: Kelch-like ECH-associated protein 1
MMP: Mitochondrial Membrane Potential
mTBM: Modified Tris Buffered Medium
Nrf2: Nuclear factor erythroid 2–related factor 2
PBS: Phosphate-Buffered Saline
PERK: PKR-like ER kinase
PVA: Polyvinyl Alcohol
PZM-3: Porcine Zygote Medium 3
qPCR: Quantitative Polymerase Chain Reaction
ROS: Reactive Oxygen Species
sXBP1: Spliced X-box Binding Protein 1
SOD: Superoxide Dismutase
TCM: Traditional Chinese Medicine
TM: Tunicamycin
UPR: Unfolded Protein Response
WB: Western Blot

## References

[1] Inhorn, M. C. & Birenbaum-Carmeli, D. Assisted reproductive technologies and culture change. *Annu. Rev. Anthropol.* **37**, 177–196. 10.1146/annurev.anthro.37.081407.085230 (2008).

[2] Grupen, C. G. The evolution of Porcine embryo in vitro production. *Theriogenology* **81**, 24–37. 10.1016/j.theriogenology.2013.09.022 (2014). 24274407 10.1016/j.theriogenology.2013.09.022

[3] Waclawik, A., Kaczmarek, M. M., Blitek, A., Kaczynski, P. & Ziecik, A. J. Embryo-maternal dialogue during pregnancy establishment and implantation in the pig. *Mol. Reprod. Dev.* **84**, 842–855 (2017). 28628266 10.1002/mrd.22835

[4] Wrenzycki, C. In vitro production of (Farm) animal embryos. *Animal Biotechnol.* **1** (Reproductive Biotechnologies), 269–304 (2018).

[5] Agarwal, A. & Majzoub, A. Role of antioxidants in assisted reproductive techniques. *World J. Mens Health*. **35**, 77–93. 10.5534/wjmh.2017.35.2.77 (2017). 28497913 10.5534/wjmh.2017.35.2.77 PMC5583374

[6] Guerin, P., Mouatassim, E., Menezo, Y. & S. & Oxidative stress and protection against reactive oxygen species in the pre-implantation embryo and its surroundings. *Hum. Reprod. Update*. **7**, 175–189. 10.1093/humupd/7.2.175 (2001). 11284661 10.1093/humupd/7.2.175

[7] Choi, S. et al. Amplification of Porcine SRY gene for sex determination. *Asian-Australasian J. Anim. Sci.* **22**, 1107–1112 (2009).

[8] Zhang, J. et al. ROS and ROS-Mediated cellular signaling. *Oxid. Med. Cell. Longev.* **2016**, 4350965. 10.1155/2016/4350965 (2016). 10.1155/2016/4350965 PMC4779832 26998193

[9] Mittler, R. R. O. S., Are & Good *Trends Plant Sci.* **22**, 11–19, doi:10.1016/j.tplants.2016.08.002 (2017). 27666517 10.1016/j.tplants.2016.08.002

[10] May-Panloup, P., Boguenet, M., Hachem, E., Bouet, H., Reynier, P. & P.-E. & Embryo and its mitochondria. *Antioxidants* **10**, 139 (2021). 33498182 10.3390/antiox10020139 PMC7908991

[11] Ufer, C., Wang, C. C., Borchert, A., Heydeck, D. & Kuhn, H. Redox control in mammalian embryo development. *Antioxid. Redox. Signal.* **13**, 833–875. 10.1089/ars.2009.3044 (2010). 20367257 10.1089/ars.2009.3044

[12] Hao, Y. et al. Apoptosis in parthenogenetic preimplantation Porcine Embryos1. *Biol. Reprod.* **70**, 1644–1649. 10.1095/biolreprod.103.026005 (2004). 14766725 10.1095/biolreprod.103.026005

[13] Li, C., Ji, K. B., Choi, H. Y., Liu, H. & Kim, M. Schisandrin B enhances embryo competence and potentially mitigates endoplasmic reticulum stress during porcine preimplantation development. 10.1016/j.theriogenology.2024.02.031 38460201

[14] Lan, M. et al. Melatonin ameliorates Ochratoxin A-induced oxidative stress and apoptosis in Porcine oocytes. *Environ. Pollut*. **256**, 113374. 10.1016/j.envpol.2019.113374 (2020). 31672358 10.1016/j.envpol.2019.113374

[15] Fang, X. et al. Vitamin C enhances Porcine cloned embryo development and improves the derivation of embryonic stem-like cells. *Reprod. Biol.* **22**, 100632 (2022). 35334451 10.1016/j.repbio.2022.100632

[16] Shen, X., Zhang, K. & Kaufman, R. J. The unfolded protein response–a stress signaling pathway of the Endoplasmic reticulum. *J. Chem. Neuroanat.* **28**, 79–92. 10.1016/j.jchemneu.2004.02.006 (2004). 15363493 10.1016/j.jchemneu.2004.02.006

[17] Read, A. & Schroder, M. The unfolded protein response: an overview. *Biology (Basel)*. **10**, 384. 10.3390/biology10050384 (2021). 33946669 10.3390/biology10050384 PMC8146082

[18] Lin, T. et al. Endoplasmic reticulum (ER) stress and unfolded protein response (UPR) in mammalian oocyte maturation and preimplantation embryo development. *Int. J. Mol. Sci.*10.3390/ijms20020409 (2019). 10.3390/ijms20020409 PMC6359168 30669355

[19] Victor, P., Sarada, D. & Ramkumar, K. M. Crosstalk between Endoplasmic reticulum stress and oxidative stress: focus on protein disulfide isomerase and Endoplasmic reticulum oxidase 1. *Eur. J. Pharmacol.* **892**, 173749. 10.1016/j.ejphar.2020.173749 (2021). 33245896 10.1016/j.ejphar.2020.173749

[20] Xu, H. Y. et al. ETCM: an encyclopaedia of traditional Chinese medicine. *Nucleic Acids Res.* **47**, D976–D982. 10.1093/nar/gky987 (2019). 30365030 10.1093/nar/gky987 PMC6323948

[21] Azietaku, J. T. et al. A review of the ethnopharmacology, phytochemistry and pharmacology of *Notopterygium incisum*. 10.1016/j.jep.2017.03.022 28336469

[22] Ma, A., Wang, X., Gong, X., Zhao, X. & Zhong, X. Protective effect of anti-abortive herbal medicine on embryo implantation and the changes of serum progesterone, IFN-γ and IL-4 in cows after artificial insemination. *J. Med. Plants Res.* **6**, 383–390 (2012).

[23] GU, Z., HATTORI, Z. H. A. N. G. D. Y. A. N. G. X., NAMBA, T. & M. & Isolation of two new coumarin glycosides from notopterygium Forbesii and evaluation of a Chinese crude drug, Qiang-Huo, the underground parts of N. incisum and N. Forbesii, by high-performance liquid chromatography. *Chem. Pharm. Bull.* **38**, 2498–2502 (1990). 10.1248/cpb.38.2498 2285981

[24] Al-Majedy, Y., Al-Amiery, A., Kadhum, A. A. & BakarMohamad, A. Antioxidant activity of coumarins. *Syst. Reviews Pharm.* **8**, 24 (2017).

[25] Matos, J. Heterocyclic antioxidants in nature: coumarins. *Curr. Org. Chem.* **21**, 311–324 (2017).

[26] Hassanein, E. H., Sayed, A. M., Hussein, O. E. & Mahmoud, A. M. Coumarins as modulators of the Keap1/Nrf2/ARE signaling pathway. *Oxidative Med. Cell. Longev.* **2020**, 1675957 (2020). 10.1155/2020/1675957 PMC7196981 32377290

[27] Zhang, P. & Yang, X. W. Studies on chemical constituents in roots and rhizomes of notopterygium incisum. *Zhongguo Zhong Yao Za zhi= Zhongguo Zhongyao Zazhi= China J. Chin. Materia Med.* **33**, 2918–2921 (2008). 19294850

[28] Parcheta, M. et al. Recent developments in effective antioxidants: the structure and antioxidant properties. *Materials* **14**, 1984 (2021). 33921014 10.3390/ma14081984 PMC8071393

[29] Chand, K. et al. A Review on Antioxidant Potential of Bioactive Heterocycle Benzofuran: Natural and synthetic derivatives. 10.1016/j.pharep.2016.11.007 28171830

[30] Egbujor, M. C., Buttari, B., Profumo, E., Telkoparan-Akillilar, P. & Saso, L. An overview of NRF2-activating compounds bearing α, β-unsaturated moiety and their antioxidant effects. *Int. J. Mol. Sci.* **23**, 8466 (2022). 35955599 10.3390/ijms23158466 PMC9369284

[31] Su, T. et al. New Benzofuran neolignans with neuroprotective activity from phyllanthodendron Breynioides. *Nat. Prod. Res.* **37**, 3798–3805 (2023). 36469675 10.1080/14786419.2022.2153454

[32] Ong, G. & Logue, S. E. Unfolding the interactions between Endoplasmic reticulum stress and oxidative stress. *Antioxid. (Basel)*. **12**, 981. 10.3390/antiox12050981 (2023). 10.3390/antiox12050981 PMC10215201 37237847

[33] Karna, K. K., Choi, B. R., Kim, M. J., Kim, H. K. & Park, J. K. The effect of schisandra chinensis baillon on Cross-Talk between oxidative Stress, Endoplasmic reticulum Stress, and mitochondrial signaling pathway in testes of Varicocele-Induced SD rat. *Int. J. Mol. Sci.* **20**, 5785. 10.3390/ijms20225785 (2019). 31744253 10.3390/ijms20225785 PMC6888522

[34] Bull, V. H. & Thiede, B. Proteome analysis of tunicamycin-induced ER stress. *Electrophoresis* **33**, 1814–1823. 10.1002/elps.201100565 (2012). 22740470 10.1002/elps.201100565

[35] Lim, E. J., Heo, J. & Kim, Y. H. Tunicamycin promotes apoptosis in leukemia cells through ROS generation and downregulation of survivin expression. *Apoptosis* **20**, 1087–1098. 10.1007/s10495-015-1135-z (2015). 26022098 10.1007/s10495-015-1135-z

[36] Wang, H. et al. Tunicamycin-induced unfolded protein response in the developing mouse brain. *Toxicol. Appl. Pharmcol.* **283**, 157–167 (2015). 10.1016/j.taap.2014.12.019 PMC4361256 25620058

[37] Lin, T. et al. Tauroursodeoxycholic acid improves pre-implantation development of Porcine SCNT embryo by Endoplasmic reticulum stress Inhibition. *Reprod. Biol.* **16**, 269–278. 10.1016/j.repbio.2016.10.003 (2016). 27765486 10.1016/j.repbio.2016.10.003

[38] Li, C., Oh, H. J., Liu, H. & Kim, M. K. Schisandrin B protects Boar spermatozoa against oxidative damage and increases their fertilization ability during in vitro storage. *Theriogenology* **198**, 194–201. 10.1016/j.theriogenology.2022.12.041 (2023). 36592517 10.1016/j.theriogenology.2022.12.041

[39] Li, C. et al. Atractylenolide Ⅲ partially alleviates tunicamycin-induced damage in Porcine oocytes during in vitro maturation by reducing oxidative stress. *Anim. Reprod. Sci.* **273**, 107761. 10.1016/j.anireprosci.2024.107761 (2025). 39765131 10.1016/j.anireprosci.2024.107761

[40] Park, A. et al. Effect of passage number of conditioned medium collected from equine amniotic fluid mesenchymal stem cells: Porcine oocyte maturation and embryo development. *Int. J. Mol. Sci.* **23**, 6569. 10.3390/ijms23126569 (2022). 35743012 10.3390/ijms23126569 PMC9224282

[41] Lee, S. M. et al. Knock-in of enhanced green fluorescent protein or/and human fibroblast growth factor 2 gene into β-Casein gene locus in the Porcine fibroblasts to produce therapeutic protein. *Asian-Australas J. Anim. Sci.* **27**, 1644–1651. 10.5713/ajas.2014.14222 (2014). 25358326 10.5713/ajas.2014.14222 PMC4213711

[42] Kim, Y. J. et al. Sirtuin 3 is essential for host defense against Mycobacterium abscessus infection through regulation of mitochondrial homeostasis. *Virulence* **11**, 1225–1239. 10.1080/21505594.2020.1809961 (2020). 32835604 10.1080/21505594.2020.1809961 PMC7549921

[43] Kozawa, M., Fukumoto, M., Matsuyama, Y. & Baba, K. Chemical Studies on the Constituents of the Chinese Crude Drug Quiang Huo". *Chem. Pharm. Bull.* **31**, 2712–2717. 10.1248/cpb.31.2712 (1983)

[44] Liu, G. Q., Dong, J., Wang, H., Hashi, Y. & Chen, S. Z. Comparison of two species of notopterygium by High-Performance liquid Chromatography—Photodiode array Detection—Electrospray ionization tandem mass spectrometry. *Eur. J. Mass Spectrom.* **18**, 59–69. 10.1255/ejms.1169 (2012). 10.1255/ejms.1169 22792615

[45] Lucas, C. G. et al. Effects of RAD51-stimulatory compound 1 (RS-1) and its vehicle, DMSO, on pig embryo culture. 10.1016/j.reprotox.2021.08.002 PMC8511169 34407461

[46] Wang, C. R. et al. Chrysoeriol improves the early development potential of Porcine oocytes by maintaining lipid homeostasis and improving mitochondrial function. *Antioxid. (Basel)*. **13**, 122. 10.3390/antiox13010122 (2024). 10.3390/antiox13010122 PMC10812720 38275647

[47] Handy, D. E. & Loscalzo, J. Redox regulation of mitochondrial function. *Antioxid. Redox Signal.* **16**, 1323–1367. 10.1089/ars.2011.4123 (2012). 22146081 10.1089/ars.2011.4123 PMC3324814

[48] Sarsour, E. H., Kumar, M. G., Chaudhuri, L., Kalen, A. L. & Goswami, P. C. Redox control of the cell cycle in health and disease. *Antioxid. Redox Signal.* **11**, 2985–3011. 10.1089/ars.2009.2513 (2009). 19505186 10.1089/ars.2009.2513 PMC2783918

[49] Ali, I. et al. Reduced glutathione alleviates tunicamycin-induced Endoplasmic reticulum stress in mouse preimplantation embryos. *J. Reprod. Dev.* **64**, 15–24 (2018). 29081452 10.1262/jrd.2017-055 PMC5830354

[50] Park, H. J. et al. Melatonin improves the meiotic maturation of Porcine oocytes by reducing Endoplasmic reticulum stress during in vitro maturation. *J. Pineal Res.* **64**, e12458 (2018). 29149522 10.1111/jpi.12458 PMC5814851

[51] Kim, J. S. et al. Tauroursodeoxycholic acid enhances the pre-implantation embryo development by reducing apoptosis in pigs. *Reprod. Domest. Anim.* **47**, 791–798 (2012). 22151574 10.1111/j.1439-0531.2011.01969.x

[52] Lu, S. C. Glutathione synthesis. *Biochim. Et Biophys. Acta (BBA) - Gen. Subj.* **1830**, 3143–3153 (2013). 10.1016/j.bbagen.2012.09.008 PMC3549305 22995213

[53] Lin, J. & Wang, L. Oxidative stress in oocytes and embryo development: implications for in vitro systems. *Antioxid. Redox. Signal.* **34**, 1394–1406 (2021). 10.1089/ars.2020.8209 33115254

[54] Millare, B., O’Rourke, B. & Trayanova, N. Hydrogen peroxide diffusion and scavenging shapes mitochondrial network instability and failure by sensitizing ROS-induced ROS release. *Sci. Rep.* **10**, 15758. 10.1038/s41598-020-71308-z (2020). 32978406 10.1038/s41598-020-71308-z PMC7519669

[55] Bonora, M. et al. Physiopathology of the permeability transition pore: molecular mechanisms in human pathology. *Biomolecules* **10**, 998 (2020). 32635556 10.3390/biom10070998 PMC7408088

