# Supplementary material for: Protective effects of Demethylfuropinnarin on porcine pre-implantation embryos under Tunicamycin-induced oxidative stress and endoplasmic reticulum stress during in vitro culture
Source: Sci Rep. 2026 Feb 5;16:7408. doi: 10.1038/s41598-026-38755-6 (PMC12929566; doi:10.1038/s41598-026-38755-6)
Supplement: Supplementary file 1 — Supplementary Material 1 [file 41598_2026_38755_MOESM1_ESM.pdf]

Materials and Methods

All chemicals and reagents, unless specified otherwise, were obtained from Sigma-Aldrich (St. Louis, MO, USA). All procedures involving the use of ovaries and semen collection were approved by the Institutional Animal Care and Use Committee (IACUC) of Chungnam National University (approval no. 202103A-CNU-002).

In vitro maturation (IVM), in vitro fertilization (IVF), and in vitro culturing (IVC)

Ovaries from prepubertal gilts were collected from a local slaughterhouse and transported to the laboratory at 37 °C in saline supplemented with 75 mg/mL penicillin G and 50 mg/mL streptomycin sulfate. Follicles ranging from 3 to 8 mm in diameter were aspirated using an 18-gauge needle attached to a 10 mL disposable syringe. Cumulus-oocyte complexes (COCs) with at least three layers of compact cumulus cells and uniformly granulated ooplasm were selected. The selected COCs were washed three times in maturation medium, which consisted of TCM-199 supplemented with 2.5 mM fructose, 0.4 mM L-cysteine, 1 mM sodium pyruvate, 0.13 mM kanamycin, 10% (v/v) porcine follicular fluid, 10 ng/mL epidermal growth factor, 10 IU/mL pregnant mare serum gonadotropin (MSD Animal Health, Kenilworth, NJ, USA), and 10 IU/mL human chorionic gonadotropin (Roche Diagnostics, Mannheim, Germany, Kenilworth, NJ, USA). The COCs were incubated at 38.5 °C under 5% CO_2 for 22 h, followed by an additional 22 h in hormone-free maturation medium (IVM medium without eCG and hCG) under the same conditions.

After 44 hours of in vitro maturation, cumulus cells surrounding the cumulus-oocyte complexes (COCs) were removed by incubation in Tyrode's Lactate HEPES-buffered medium

(NCSU-W) supplemented with 1 mg/mL hyaluronidase. The composition of the medium was as follows: 114 mM NaCl, 3.2 mM KCl, 2 mM $NaHCO_3$, 0.4 mM NaH_2PO_4, 10 mM sodium lactate, 0.5 mM $MgCl_2 \cdot 6H_2O$, 10 mM HEPES, 2 mM $CaCl_2 \cdot 2H_2O$, 0.01% PVA, 12 mM sorbitol, and 0.25 mM sodium pyruvate. The pH of the medium was adjusted to 7.2–7.4, with an osmotic pressure of 295–310 mOsm. Oocytes exhibiting a visible first polar body were selected and subsequently washed three times in modified Tris-buffered medium (mTBM), which contained 113.1 mM NaCl, 3.0 mM KCl, 7.5 mM $CaCl_2 \cdot 2H_2O$, 20.0 mM Tris base, 11.0 mM glucose, 5.0 mM sodium pyruvate, 1.0 mM caffeine, 10 mg/mL gentamicin, and 0.2% BSA. The pH of mTBM was adjusted to 7.4, with an osmotic pressure of 290–300 mOsm. Washed oocytes were then transferred to fresh mTBM drops for fertilization. Semen was obtained from Darby Genetics Inc. (Seodong-daero, Iljuk-myeon, Anseong, Gyeonggi-do, Republic of Korea). Ejaculates from seven different Duroc boars were used across experiments, with the number of boars varying depending on the specific experimental design. Semen collection was performed via the hand-grip method, and fresh ejaculates were immediately diluted in a commercial extender provided by the supplier. The extended semen was transported to the laboratory at 17–18°C. Only semen samples with ≥80% total motility and ≤15% morphologically abnormal spermatozoa were used. The sperm pellets were washed and diluted in mTBM to a concentration of 2×10^6 sperm/mL. Diluted sperm were added to the fertilization drops containing the oocytes to achieve a final concentration of 0.25×10^6 sperm/mL. Oocytes and sperm were co-incubated for 6 hours at 38.5°C under 5% CO_2 and 100% humidity.

After fertilization, embryos were washed three times and transferred into PZM-3 medium (bicarbonate-buffered porcine zygote medium 3), consisting of: 10.8 mM NaCl, 0.04 mM

$MgSO_4$, 1 mM KCl, 0.35 mM KH_2CO_3, 0.13 mM kanamycin, 0.2 mM Na-pyruvate, 2.0 mM Ca-lactate, 1.0 mM L-glutamine, 5.0 mM hypotaurine, and 3 mg/mL BSA (pH 7.3; 282–286 mOsm). Embryo cleavage and blastocyst formation rates were assessed on Day 2 and Day 7 after IVF, respectively. As shown in Supplementary Figure 1, the calculation of the blastocyst rate included only Expanded Blastocysts, Hatching Blastocysts, and Hatched Blastocysts.

Analysis of ROS levels, GSH levels, MMP values, mitochondrial function, ER distribution, and apoptosis levels in blastocysts

ROS, GSH, and Fe^{2+} levels were assessed using the Image-iT™ LIVE Green ROS Detection Kit (Invitrogen, Carlsbad, CA, USA) and CellTracker™ Blue CMF2HC (Invitrogen, Carlsbad, CA, USA), respectively. Blastocysts were washed three times in 0.1% (w/v) phosphate-buffered saline containing polyvinyl alcohol (PBS/PVA), and incubated in 0.1% PBS/PVA supplemented with 25 μM H_2DCF-DA (for ROS) or 10 μM CMF2HC dye (for GSH) at 38.5 °C for 30 minutes. Following incubation, embryos were washed again and fluorescence signals were detected using appropriate filters (ROS: 460 nm; GSH: 370 nm).

Mitochondrial membrane potential (MMP) levels were evaluated using the MitoProbe™ JC-1 Assay Kit (Invitrogen, Carlsbad, CA, USA). Blastocysts were washed three times in 0.1% PBS/PVA, and incubated in 0.1% PBS/PVA containing 2 μM JC-1 at 38.5 °C for 30 minutes. After staining, fluorescence was observed using a fluorescence microscope equipped with appropriate filters (J-aggregates, 580 nm and J-monomers, 460 nm). MMP levels were quantified as the ratio of red fluorescence intensity to green fluorescence intensity.

Mitochondrial function and endoplasmic reticulum (ER) distribution in porcine blastocysts were assessed using MitoTracker™ Red CMXRos and ER-Tracker™ (Thermo

Fisher Scientific, Waltham, MA, USA), respectively. Blastocysts were washed in 0.1 % PBS/PVA and stained with 250 nM MitoTracker™ Red CMXRos or 200 nM ER-Tracker™ in 0.1% PBS/PVA at 38.5 °C for 30 minutes. Following staining, embryos were washed with 0.1% PBS/PVA, and fluorescence signals were detected using a fluorescence microscope with a 580 nm filter.

Apoptotic cells in blastocysts were detected using a TUNEL assay (In Situ Cell Death Detection Kit, TMR Red, Roche Diagnostics, Mannheim, Germany). Blastocysts were fixed in 4% phosphate-buffered saline containing paraformaldehyde (PBS/PFA) for 1 hour, followed by permeabilization with 1% Triton X-100 for 30 minutes at room temperature (24 - 25 °C). After permeabilization, embryos were incubated in TUNEL reaction mixture (enzyme solution/label solution = 1:9) at 38.5 °C for 1 hour in the dark. After washing with 0.1% PBS/PVA, blastocysts were counterstained with Hoechst 33342 for 5 minutes at room temperature. After mounting the embryos on glass slides with coverslips, apoptotic nuclei (TUNEL-positive, 580 nm) and total nuclei (Hoechst-stained, 370 nm) were visualized using a fluorescence microscope.

Fluorescence Detection and Quantification in Blastocysts

All fluorescence was detected using a Leica DMi8 fluorescence microscope (Leica Microsystems CMS GmbH, Wetzlar, Germany), and images were captured with a Leica DFC7000T imaging system using Leica Application Suite X 3.7.32345 software. All fluorescence intensity analysis was conducted using ImageJ software (version 1.51, NIH, USA). For quantification, the integrated density of each blastocyst was measured within the blastocyst area. Each blastocyst was treated as a single biological replicate (n = 1). The number of stained

blastocysts per group is indicated in the corresponding figure panels, and the number of independent experimental replicates is stated in the figure legends. Quantification results are presented as relative fluorescence intensity, with the control group normalized to 1 (arbitrary units).

Western blot (WB)

Western blotting was performed to analyze the expression levels of Nrf2 and Keap1 proteins in blastocysts. The anti-Nuclear factor erythroid 2–related factor 2 (Nrf2) antibody (BS-1074R) and anti-Kelch-like ECH-associated protein 1 (Keap1) antibody (BS-3648R) were obtained from Thermo Fisher Scientific (Waltham, MA, USA), and the anti-β-actin antibody was purchased from Sigma-Aldrich (St. Louis, MO, USA). Goat anti-rabbit HRP-conjugated secondary antibody was obtained from Santa Cruz Biotechnology (Santa Cruz, CA, USA). For each group, a minimum of 40 blastocysts were collected and lysed in RIPA buffer (ELPIS Bio, Eunpyeong-gu, Seoul, South Korea) containing protease and phosphatase inhibitors. Proteins were precipitated using cold acetone, air-dried, and re-suspended in TEN buffer. Protein concentrations were determined using the Bio-Rad protein assay kit (Sigma-Aldrich, St. Louis, MO, USA), following the manufacturer's instructions. Equal amounts of protein (20–30 μg per lane) were separated by SDS-PAGE on 10% polyacrylamide gels and transferred to PVDF membranes (Bio-Rad Laboratories, Hercules, CA, USA) using a wet-transfer system. Membranes were blocked with 5% non-fat dry milk in TBST (Tris-buffered saline with 0.1% Tween-20) for 1 hour at room temperature and then incubated overnight at 4 °C with the primary antibodies (1:1,000 dilution for Nrf2,

Keap1, and β-actin). After washing with TBST, membranes were incubated with HRP-conjugated secondary antibody (1:5,000 dilution) for 1 hour at room temperature. Protein bands were visualized using an enhanced chemiluminescence detection kit (Amersham, Arlington Heights, IL, USA) and captured with the Alliance Q9 Advanced imaging system (UVItec Ltd., Cambridge, UK). Band intensities were quantified using ImageJ software (NIH, USA), and the expression levels of Nrf2 and Keap1 were normalized to β-actin. Uncropped full-length blot images are provided in Supplementary Figure S4.

Analysis of superoxide dismutase (SOD) and catalase (CAT) activity levels in blastocysts

The activities of superoxide dismutase (SOD) and catalase (CAT) were evaluated using the OxiSelect™ Superoxide Dismutase Activity Assay Kit and OxiSelect™ Catalase Activity Assay Kit, respectively (Cell Biolabs, San Diego, CA, USA), according to the manufacturers' protocols. For each assay, fifty blastocysts per group were pooled and lysed for biochemical analysis. Absorbance values were measured at 490 nm for SOD and 520 nm for CAT recorded using a Synergy™ H1 Multi-Mode Microplate Reader (BioTek Instruments, Winooski, VT, USA). The results were normalized by setting the mean value of the control group to 1 and were expressed as relative values without units.

Real-time quantitative reverse transcription polymerase chain reaction (RT-qPCR)

Total RNA was extracted from each group of blastocysts using the RNAqueous™ Micro Kit (Thermo Fisher Scientific, Waltham, MA, USA), following the manufacturer's

instructions. A total of 150–200 blastocysts per group were used for RNA extraction. RNA concentration and purity were measured using a BioSpec-nano microvolume spectrophotometer (Shimadzu Biotech, Kyoto, Japan). Only samples with an A260/A280 ratio between 1.8 and 2.1 were used for downstream analysis. Genomic DNA contamination was eliminated through on-column DNase I treatment, and all RNA samples were verified to be DNA-free via PCR-based detection. To assess RNA integrity, purified RNA samples were subjected to 1% agarose gel electrophoresis, and the presence of distinct 28S and 18S rRNA bands with an approximate 2:1 intensity ratio was taken as indicative of intact RNA. Reverse transcription was performed using the Maxime™ PreMix (iNtRON Biotechnology, Seongnam, South Korea), using 200 ng of total RNA per sample according to the manufacturer's protocol. Quantitative PCR amplification was carried out using the BIO-RAD CFX96™ real-time PCR system (Bio-Rad Laboratories, Hercules, CA, USA) with PowerUp™ SYBR™ Green Master Mix (Applied Biosystems, Foster City, CA, USA). Each 20 μL reaction contained 10 μL of SYBR Green Master Mix, 1 μL of forward primer, 1 μL of reverse primer, 2 μL of cDNA template, and 6 μL of nuclease-free water. Thermal cycling conditions were as follows: 95°C for 3 minutes, followed by 39 cycles of 95°C for 10 seconds, 60°C for 10 seconds, and 72°C for 30 seconds. A melting curve analysis from 65°C to 95°C (0.5°C increments every 5 seconds) was conducted at the end of each run to confirm product specificity. The primer sequences, amplicon sizes, and GenBank accession numbers are provided in Supplementary Table 1. All primers were designed to span exon–exon junctions to avoid amplification of genomic DNA. PCR product specificity was confirmed by both agarose gel electrophoresis and

Sanger sequencing. Non-template controls (NTC) and no reverse transcription controls (–RT) were included for each gene to ensure that no amplification resulted from contamination or genomic DNA. The mRNA expression levels of sXBP1, ATF4, GRP78, CHOP, Bcl2, and Bax were analyzed, with normalization to GAPDH, a previously validated stable reference gene in porcine embryos. GAPDH 2^{-Cq} values did not differ significantly among experimental groups (ANOVA, $P > 0.05$). Gene expression levels were quantified using the $2^{-\Delta\Delta Cq}$ method.

Statistical analysis

All data are presented as mean ± standard deviation (SD). The number of replicates and sample sizes for each experiment are provided in the corresponding figures and figure legends. Percentage data were analyzed using a beta regression model, while continuous data were analyzed using one-way analysis of variance (ANOVA) followed by Tukey's post-hoc test or Student's t-test, as appropriate. Prior to one-way ANOVA, data were assessed for normality using the Shapiro–Wilk test and for homogeneity of variances using Levene's test. When assumptions of normality or homogeneity were not met, data were subjected to arcsine square root transformation, followed by robust ANOVA. All statistical analyses were performed using R software version 4.3.2. Different superscript letters indicate statistically significant differences within experimental groups ($P < 0.05$). Asterisks denote levels of statistical significance as follows: $^{*}P < 0.05$, $^{**}P < 0.01$, and $^{***}P < 0.001$.

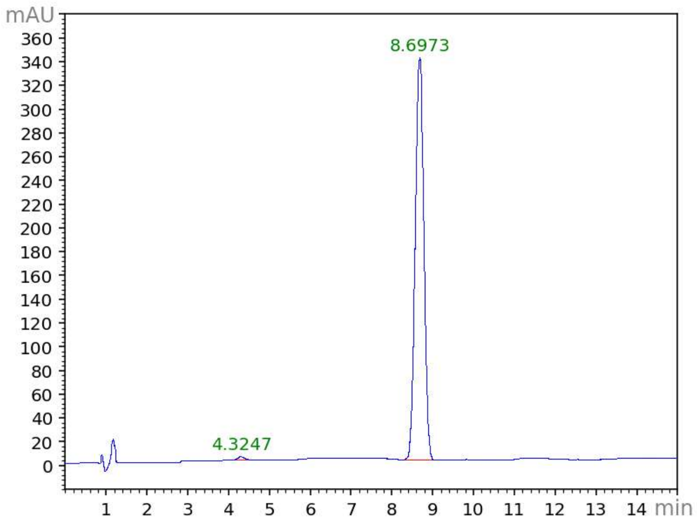

Supplementary figure 1. **HPLC chromatogram of the analyzed sample.** The main peak at approximately 8.70 min corresponds to the target compound, while the minor peak at approximately 4.32 min represents a detected impurity.

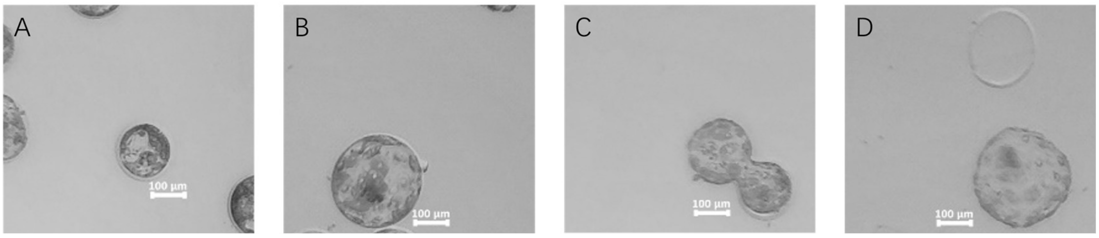

Supplementary figure 2. **Classification of Blastocysts.** Early Blastocyst (A); Expanded Blastocyst (B); Hatching Blastocyst (C); Hatched Blastocyst (D).

preliminary dose-response experiment

Effects of different concentrations of DMFP on the developmental potential of porcine embryos

The developmental potential of porcine embryos exposed to DMFP concentrations of 0, 0.1, 1, 10, and 100 mg/L was assessed, as detailed in Supplementary Figure 3 and Supplementary Table 2. The developmental status of embryos in each group is shown in Supplementary Figure 4. Embryos treated with 100 mg/L DMFP had a significantly lower cleavage rate compared to 0.1 mg/L, 1 mg/L, and 10 mg/L DMFP groups (Figure 1A, $P < 0.05$). The blastocyst rates of embryos from the 1 mg/L group displayed significantly higher rates compared to 0 mg/L and 100 mg/L groups (Figure 1B, $P < 0.05$). The blastocyst rate for embryos treated with 100 mg/L DMFP was significantly lower than other groups (Figure 1B, $P < 0.05$).

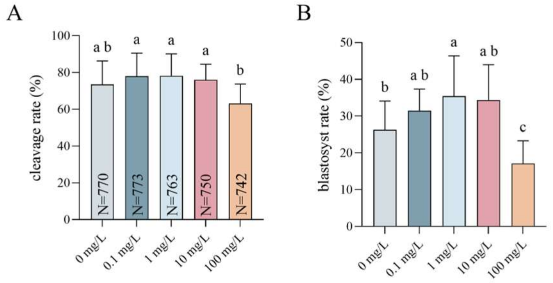

Supplementary Figure 3. **Effects of different concentrations of DMFP on the developmental potential of porcine embryos.** The cleavage rate (A) and blastocyst rate (B) of embryos treated with different concentrations (0, 0.1, 1, 10, and 100 mg/L) of DMFP. Sample sizes (N) are indicated directly within each bar of the figure. A total of 15 independent experimental replicates were performed. Different superscripts indicate statistical differences within experimental groups ($P < 0.05$).

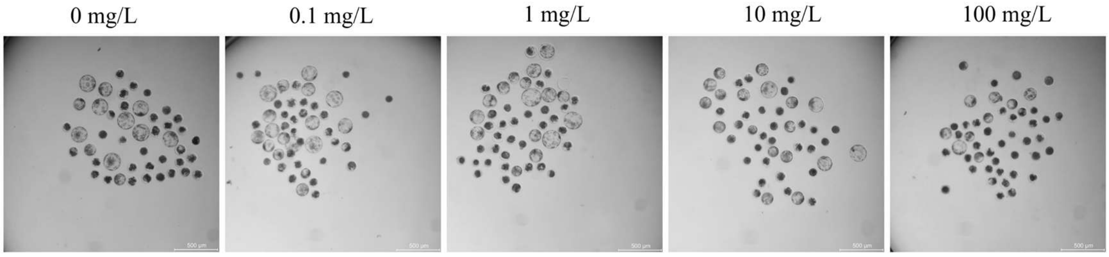

Supplementary figure 4. **The developmental status of porcine embryos exposed to DMFP concentrations of 0, 0.1, 1, 10, and 100 mg/L.** Representative images showing the morphology of embryos in each group. These images are for illustrative purposes only. Quantitative developmental data are provided in Supplementary Figure 1 and Supplementary Table 1.

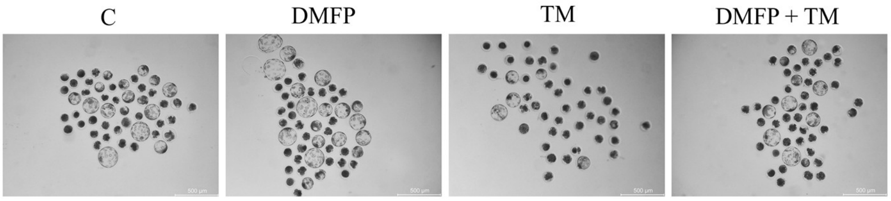

Supplementary figure 5. **The developmental status of porcine embryos from different groups.** Representative images showing the morphology of embryos in each group. These images are for illustrative purposes only. Quantitative developmental data are provided in Figure 1 and Supplementary Table 2.

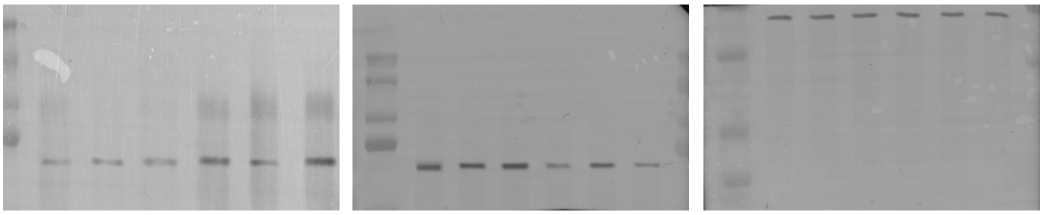

Supplementary figure 6. **Western blot images corresponding to Figure 2G.** The blots show full-length signals for Nrf2, Keap1, and β-actin, respectively. All images were captured under uniform exposure conditions and without digital enhancement.

Supplementary Table 1. Primers used for real-time qPCR

Primer Name	Accession No.	Sequence	bp
Bax-F	XM_003127290	GGTCGCGCTTTTCTACTTTG	203
Bax-R		CGATCTCGAAGGAAGTCCAG	
Bcl2-F	NM_214285.1	AATGTCTCAGAGCAACCGGG	144
Bcl2-R		GGGGCCTCAGTTCTGTTCTC	
sXBP1-F	NM_001271738.1	GGAGTTAAGACAGCGCTTGG	142
sXBP1-R		GAGATGTTCTGGAGGGGTGA	
ATF4-F	NM_001123078.1	AGTCCTTTTCGCGAGTGGG	126
ATF4-R		CTGCTGCCTCTAATACGCCA	
GRP78-F	XM_001927795.7	TGGAGGCCACATCAATCATA	146
GRP78-R		AGCGGTCAACTTCTCCTTGA	
CHOP-F	NM_001144845.1	TCTGGCTTGGCTGACTGAGGAG	139
CHOP-R		TTTCCGTTTCCTGGGTCTTCTTTGG	
GAPDH-F	NM_001206359	CCCCAACGTGTCGGTTGT	91
GAPDH-R		CTCGGACGCCTGCTTCAC	

Supplementary Table 2. Effects of Different Concentrations of DMFP on Cleavage Rate and Blastocyst Rate of Porcine embryos.

Group	embryos (n)	Cleavage rate (%[n])	Blastocyst rate (%[n])
0 mg/L	512	73.6±12.74(377) ^{a}	27.43±8.33(131) ^{b}
0.1 mg/L	516	77.89±12.44(402) ^{a}	31.58±5.86(154) ^{ab}
1 mg/L	509	78.19±12.08(396) ^{a}	35.91±9.54(167) ^{a}
10 mg/L	499	76.08±8.55(380) ^{a}	34.4±9.66(172) ^{a}
100 mg/L	485	63.35±10.40(308) ^{b}	17.02±6.19(83) ^{c}

Supplementary table 3. Effects of DMFP and TM on Cleavage Rate and Blastocyst Rate of Porcine embryos.

Group	embryos (n)	Cleavage rate (%[n])	Blastocyst rate (%[n])
C	685	74.21±11.24(521) ^{ab}	25.81±8.88(178) ^{b}
DMFP	707	75.15±11.46(530) ^{a}	33.67±12.51(237) ^{a}
TM	699	68.39±6.79(478) ^{c}	13.17±3.68(92) ^{d}
DMFP+TM	673	68.72±12.00(461) ^{bc}	18.79±5.63(126) ^{c}
